# Primary and Secondary Human Bocavirus 1 Infections in a Family, Finland

**DOI:** 10.3201/eid.1908.130074

**Published:** 2013-08

**Authors:** Alma Jula, Matti Waris, Kalle Kantola, Ville Peltola, Maria Söderlund-Venermo, Klaus Hedman, Olli Ruuskanen

**Affiliations:** Turku University Hospital, Turku, Finland (A. Jula, V. Peltola, O. Ruuskanen);; University of Turku, Turku (M. Waris);; University of Helsinki, Helsinki, Finland (K. Kantola, M. Söderlund-Venermo, K. Hedman)

**Keywords:** human bocavirus, HBoV, rhinovirus, transmission, respiratory virus, viruses, Finland

## Abstract

Human bocavirus 1 (HBoV1) was detected in a young child hospitalized for pneumonia and subsequently in his twin brother and other family members. The mother’s nasopharyngeal samples intermittently showed HBoV1 DNA; the grandmother had HBoV1 reinfection. Findings in this family lead to consideration of HBoV virulence, latency, and reactivation.

Human bocavirus 1 (HBoV1) is a frequent cause of common cold, bronchiolitis, acute wheezing, and pneumonia in children worldwide. The causative role of HBoV1 has been questioned because of HBoV1 DNA presence after primary infection and common co-infection with other respiratory viruses (*1**–**5*)*.* We report life-threatening HBoV1 pneumonia in a child and transmission within his family.

## The Cases

The index patient was a male twin who was born prematurely at 26 weeks’ gestation. He weighed 1,540 g at birth and had severe bronchopulmonary dysplasia. He was not administered pneumococcal vaccine. At the age of 16 months, he was admitted to the hospital for evaluation and treatment of wheezing. Rhinorrhea and cough had been present for 12 days. No fever was recorded at admission. The child had a heart rate of 170 beats/min (reference range 75–130 beats/min) and a respiratory rate of 50 breaths/min (reference range 25–30 breaths/min). He experienced severe respiratory distress and was transferred to the intensive care unit. A chest radiograph showed bilateral pulmonary infiltrations and atelectasis of the upper right lobe (Figure). His serum C-reactive protein level was 1 mg/L, and his leukocyte count was 19.6 ×10^9^/L. He was treated with intravenous cefuroxime, clarithromycin, and methylprednisolone. Tracheal intubation and bronchoalveolar lavage (BAL) were performed. Cultures of blood and BAL sample did not grow bacteria.

**Figure:**
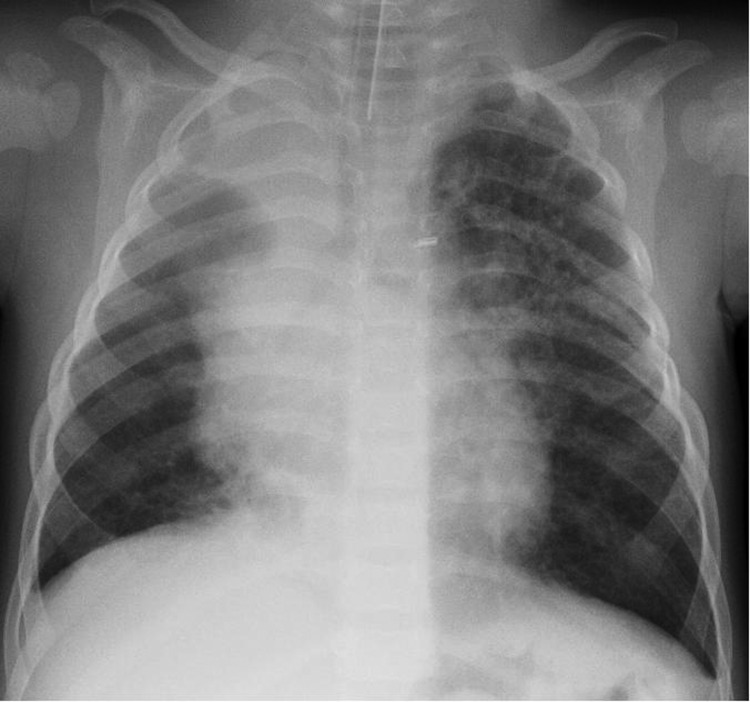
Chest radiograph of the index patient, a 16-month-old boy in Finland with human bocavirus 1 pneumonia, on day 2 of hospitalization. Bilateral pulmonary infiltrations and atelectasis of the upper right lobe can be seen.

After a week, ventilator-associated pneumonia developed in the child. His serum C-reactive protein level rose to 68 mg/L; intravenous vancomycin and meropenem treatment were initiated. *Pseudomonas aeruginosa* was identified in a second BAL culture. The child was extubated after 3 weeks, and after 5 weeks of hospitalization, he recovered completely.

The patient’s twin brother had recovered from a common cold, which had started 2 weeks before the patient was hospitalized. Two days after the patient was admitted, his brother contracted another mild respiratory infection lasting 4 days. The 34-year-old mother remained asymptomatic, but the 58-year-old grandmother, who stayed in the household daily for many hours, had rhinitis. All family members were tested for respiratory viruses.

Nasopharyngeal aspirate (NPA) collected from the index patient was negative by PCR for influenza A and B, parainfluenza type 1–3 viruses, adenovirus, human metapneumovirus, respiratory syncytial virus, enteroviruses, and coronavirus types 229E, OC 43, NL63, and HKU1. PCR revealed a low load of human rhinovirus (HRV) RNA and a much higher load of HBoV1 DNA (Table). The index patient had an HBoV1 infection proven by detection of HBoV1 DNA in high copy numbers in NPA and BAL samples, as well as in serum. The DNA levels gradually decreased until they were not detectable (Table). PCR showed the index patient’s serum samples to be HBoV1-positive for 4 weeks and nasal mucus samples to be positive for at least 3 months. All samples were negative for HBoV2–4 DNA. The child had an HBoV1-specific IgM response and a >4-fold increase in HBoV1 IgG. The avidity of the IgG was initially low and then matured, but IgM declined and were undetectable by the end of the 3-month follow-up period. 

**Table:** HBoV1 DNA, other respiratory viruses, and HBoV antibodies in family members during 7 months, Finland*

Patient and no. days after hospital admission of index patient	Symptoms of acute infection	HBoV1 DNA in NPA, copies/mL	HBoV1 DNA in serum, copies/mL†	HBoV1 IgM‡	HBoV IgG avidity	HBoV1 IgG‡	HBoV2 IgG‡	HBoV3 IgG‡§	Other viruses detected in NPA by PCR
Index									
1	Yes	1.6 × 10^10^	NT	NT	NT	NT	NT	NT	None
4	Yes	4.1 × 10^8^	3,000	2.148	2.0	1.034	0	0	HRV
9	Yes	3.0 × 10^5^	1,400	2.089	2.0	3.127	0	0.034	HRV negative
29	Yes	300	440	1.613	9.7	3.028	0.060	0	HRV negative
64	No	NT	Negative	0.154	17.1	3.426	0.050	0	NT
74	No	5,400	NT	NT	NT	NT	NT	NT	None
109	Yes	600	Negative	0.040	31.3	3.221	0	0	PIV1
185	ND	Negative	NT	NT	NT	NT	NT	NT	NT
232	Yes	Negative	NT	NT	NT	NT	NT	NT	AdV
Twin brother									
5	Yes	6,300	NT	NT	NT	NT	NT	NT	HRV
14	ND	7,800	NT	NT	NT	NT	NT	NT	HRV
29	ND	3,900	NT	NT	NT	NT	NT	NT	HRV
64	No	NT	Negative	0.022	26.9	3.305	0	0	None
74	No	Negative	NT	NT	NT	NT	NT	NT	None
109	Yes	99,000	Negative	0.011	38.2	3.242	0	0	PIV1
232	Yes	5,700	NT	NT	NT	NT	NT	NT	AdV
Mother									
5	No	Negative	NT	NT	NT	NT	NT	NT	HRV
13	ND	Negative	NT	0.013	50.2	0	0.509	0.307	HRV negative
30	No	Negative	NT	0.019	45.0	0.001	0.542	0.360	HRV negative
64	No	Negative	NT	0.019	48.1	0.012	0.480	0.259	NT
109	No	300	NT	0.032	50.2	0	0.861	0.585	PIV1
Grandmother									
5	Yes	5,100	NT	NT	NT	NT	NT	NT	None
13	ND	22,000	960	0.011	58.0	0.395	0.542	0.016	HRV negative
64	ND	NT	Negative	0.012	25.4	2.731	1.470	0	None
74	No	3,600	NT	NT	NT	NT	NT	NT	HRV negative
109	Yes	300	Negative	0.023	33.5	2.674	1.834	0	PIV1, HRV

*HBoV1, human bocavirus 1; NPA, nasopharyngeal aspirate; NT, not tested; HRV, human rhinovirus; HRV negative, tested by monoplex PCR only; PIV1, parainfluenzavirus type 1; ND, no data available; AdV, adenovirus; none, negative by multiplex PCR. †All serum samples were negative for HBoV2–4 DNA (*6*). ‡HBoV antibodies after heterologous antigen competition are shown as absorbance values at 492 nm, cutoff 0.13 (*7*)*.* §IgG avidity was calculated as the ratio of 2 IgG end-point titers (urea positive/urea negative) as described (*8*). Results obtained with unblocked enzyme immunoassay and confirmed with specific competition enzyme immunoassays.

The twin brother’s NPA was positive for HBoV1 DNA for 6 weeks. Two months later, his test results were HBoV1 IgM–negative but exhibited HBoV1 IgG at a high level and of high avidity. The children’s mother, who was asymptomatic, lacked HBoV1–4 DNA in serum but had intermittent low HBoV1 DNA loads in NPA. She had neither HBoV1-specific IgM nor IgG but exhibited a stable level of HBoV2-specific IgG of high avidity. NPA collected from the grandmother was intermittently HBoV1 DNA–positive twice during the first 9 days and twice thereafter. On day 13 of her grandson’s hospitalization, her serum was HBoV1 DNA–positive; on follow-up, she had an increase of high-avidity HBoV1 IgG but no IgM response.

The index patient was tested for HRV RNA 8 times during hospitalization. His results were positive (1.5 × 10^5^ copies/mL) once, on day 4 after admission. His mother’s test result was positive (1.9 × 10^4^ copies/mL) on day 5. The twin brother's test results were positive (5.4 × 10^7^ – 1,500 copies/mL) on days 5, 14, and 29, indicating he had an acute HRV infection. Three months after this episode of HBoV1 infection, all 4 family members had symptomatic parainfluenza type 1 virus infections, and clinical samples for the twin brother were again positive for HBoV1 DNA. Four months later, both brothers had an adenovirus infection, and the twin brother’s test result was again positive for HBoV1.

For each family member tested, quantitative PCR for HBoV1 in NPA was performed as described (*9*)*.* HBoV1–4 DNA in serum was measured by multiplex and singleplex PCRs (*6*). For other respiratory viruses, qualitative multiplex PCR (SeeplexRV12, Seegene, Seoul, South Korea) and quantitative PCR methods were used (*10*)*.* Biotinylated HBoV1–3 VP2 virus-like particles were applied as antigens in enzyme immunoassays for measurement of HBoV-specific IgM, IgG, and IgG avidity (*8**,**11*). For removal of cross-reacting heterologous HBoV antibodies, virus-like particle–based competition assays were used (*7*).

## Conclusions

The index patient had life-threatening pneumonia associated with acute HBoV1 infection and was at high risk for complications from the infection. Cases of severe lower respiratory tract HBoV1 infection in 3 other young children have been reported (*12**–**14*). Their illnesses were characterized by severe respiratory distress associated with pneumothorax and pneumomediastinum; 2 of them required mechanical ventilation, and 1 required extracorporeal membrane oxygenation. The children fully recovered.

This article adds to evidence of HBoV1 virulence by describing probable transmission of HBoV1 infection within a family. The index patient had a severe HBoV1 infection, and his twin brother had a mild infection with low viral load. This finding is supported by serologic studies 2–3 months later when the index patient had HBoV1 IgG of stable level and high avidity with slight maturation. The mother seems not to have acquired an HBoV1 infection. She was nonviremic, yet once showed borderline HBoV1 DNA positivity in NPA, reflecting either replicative infection or noninfective mucosal contamination. HBoV2 IgG in the mother appears to have preceded her exposure to HBoV1, which failed to raise specific HBoV1 IgG, possibly because of a phenomenon known as original antigenic sin (*15*). The lack of symptoms in the mother further suggests that her preexisting HBoV2 IgG may have been cross-protective. Of note, despite the presence of preexisting HBoV1 IgG, the grandmother had a symptomatic HBoV1 infection, which was probably caused by reinfection characterized by low-load viremia and simultaneous increases in HBoV1 and HBoV2 IgG.

During the acute phase of HBoV1 infection in the index patient, HRV RNA was identified in the NPA of 3 family members, an observation similar to that of many studies (*1*). The effect of HRV on the symptoms in this family cannot be reliably judged, but it is possible that the high-risk index patient’s severe illness was partly caused by the simultaneous or preceding HRV infection. During follow up, all family members experienced parainfluenza type 1 virus infections, and the children also had adenovirus infections. In both instances, a recurrence of HBoV1 DNA was observed in NPA from the twin brother. These observations suggest that other respiratory viruses might reactivate HBoV1 from latency, as has been seen with several other DNA viruses, or that they alter the intranasopharyngeal environment and release persisting HBoV.

Our observations raise several questions that warrant further study. First, can the presence of HBoV1 in the NPA represent only passive, nonreplicating mucosal contamination from a family member? Second, can HBoV1 establish true latency, and if so, can other respiratory viruses reactivate the virus? Third, how often does HBoV1 cause symptomatic reinfections? Last, does immunity to HBoV2 or HBoV3 cross-protect against HBoV1 and vice versa?

## References

[1] Jartti T, Hedman K, Jartti L, Ruuskanen O, Allander T, Söderlund-Venermo M. Human bocavirus–the first 5 years. Rev Med Virol. 2012;22:46–64. 10.1002/rmv.720 22038931

[2] Meriluoto M, Hedman L, Tanner L, Simell V, Mäkinen M, Simell S, Association of human bocavirus 1 infection with respiratory disease in childhood follow-up study, Finland. Emerg Infect Dis. 2012;18:264–71. 10.3201/eid1802.111293 22305021 PMC3310460

[3] Longtin J, Bastien M, Gilca R, Leblanc E, de Serres G, Bergeron MG, Human bocavirus infections in hospitalized children and adults. Emerg Infect Dis. 2008;14:217–21. 10.3201/eid1402.070851 18258113 PMC2600186

[4] Martin ET, Fairchok MP, Kuypers J, Magaret A, Zerr DM, Wald A, Frequent and prolonged shedding of bocavirus in young children attending daycare. J Infect Dis. 2010;201:1625–32. 10.1086/652405 20415535 PMC2862123

[5] Christensen A, Nordbø SA, Krokstad S, Rognlien AG, Døllner H. Human bocavirus in children: mono-detection, high viral load and viraemia are associated with respiratory tract infection. J Clin Virol. 2010;49:158–62. 10.1016/j.jcv.2010.07.016 20833582 PMC7108378

[6] Kantola K, Sadeghi M, Antikainen J, Kirveskari J, Delwart E, Hedman K, Real-time quantitative PCR detection of four human bocaviruse**s.** J Clin Microbiol. 2010;48:4044–50. 10.1128/JCM.00686-10 20844210 PMC3020864

[7] Kantola K, Hedman L, Arthur J, Alibeto A, Delwart E, Jartti T, Seroepidemiology of human bocaviruses 1–4. J Infect Dis. 2011;204:1403–12. 10.1093/infdis/jir525 21921203 PMC3988444

[8] Hedman L, Söderlund-Venermo M, Jartti T, Ruuskanen O, Hedman K. Dating of human bocavirus infection with protein-denaturing IgG-avidity assays– secondary immune activations are ubiquitous in immunocompetent adults. J Clin Virol. 2010;48:44–8. 10.1016/j.jcv.2010.02.003 20227338

[9] Koskenvuo M, Möttönen M, Waris M, Allander T, Salmi TT, Ruuskanen O. Human bocavirus in children with acute lymphoblastic leukemia. Eur J Pediatr. 2008;167:1011–5. 10.1007/s00431-007-0631-8 18038236 PMC7086950

[10] Peltola V, Waris M, Österback R, Susi P, Ruuskanen O, Hyypiä T. Rhinovirus transmission within families with children: incidence of symptomatic and asymptomatic infections. J Infect Dis. 2008;197:382–9. 10.1086/525542 18248302

[11] Söderlund-Venermo M, Lahtinen A, Jartti T, Hedman L, Kemppainen K, Lehtinen P, Clinical assessment and improved diagnosis of bocavirus-induced wheezing in children, Finland. Emerg Infect Dis. 2009;15:1423–30. 10.3201/eid1509.090204 19788810 PMC2819894

[12] Ursic T, Steyer A, Kopriva S, Kalan G, Krivec U, Petrovec M. Human bocavirus as the cause of a life-threatening infection. J Clin Microbiol. 2011;49:1179–81. 10.1128/JCM.02362-10 21227992 PMC3067724

[13] Edner N, Castillo-Rodas P, Falk L, Hedman K, Söderlund-Venermo M, Allander T. Life-threatening respiratory tract disease with human bocavirus-1 infection in a 4-year-old child. J Clin Microbiol. 2012;50:531–2. 10.1128/JCM.05706-11 22135260 PMC3264148

[14] Körner RW, Söderlund-Venermo M, van Koningsbruggen-Rietschel S, Kaiser R, Malecki M, Schildgen O. Severe human bocavirus infection, Germany. Emerg Infect Dis. 2011;17:2303–5. 10.3201/eid1712.110574 22172367 PMC3311181

[15] Morens DM, Burke DS, Halstead SB. The wages of original antigenic sin. Emerg Infect Dis. 2010;16:1023–4. 10.3201/eid1606.100453 20507764 PMC3086238

